# Development and validation of a competency-based ladder pathway for AI literacy enhancement among higher vocational students

**DOI:** 10.1038/s41598-025-15202-6

**Published:** 2025-08-14

**Authors:** Litian Hong

**Affiliations:** School of Smart Tourism, Business Administration, Zhejiang Technical Institute of Economics, Hangzhou, 310018 Zhejiang China

**Keywords:** AI literacy, Competency-based education, Ladder development pathway, Higher vocational education, Artificial intelligence, Workforce development, Computer science, Information technology

## Abstract

The rapid integration of artificial intelligence across industries necessitates systematic AI literacy development in higher vocational education to prepare students for AI-driven professional environments. This study develops and validates a comprehensive competency-based ladder development pathway specifically designed to enhance AI literacy among vocational students. The research employs a mixed-methods approach combining theoretical framework construction, empirical investigation, and practical implementation validation. The three-tier pathway model integrates foundational cognitive, skills application, and comprehensive innovation layers to address diverse learning needs while maintaining progression standards. Through empirical investigation involving 2850 students across 15 institutions, the study identifies distinct learner profiles and competency deficits, informing personalized development strategies. The validation experiment with 420 participants demonstrates significant improvements across all competency dimensions, with overall AI literacy gains of 56.0% and sustained retention rates exceeding 85% at six-month follow-up. The innovative pedagogical approaches incorporate project-driven learning, experiential methodologies, and hybrid delivery models to optimize competency development. The comprehensive evaluation framework provides robust assessment tools that balance formative and summative approaches while maintaining alignment with industry standards. Results indicate that students in the ladder pathway intervention achieved 34.7% higher cognitive assessment scores, 42.3% superior performance on skills application tasks, and 28.9% better innovation competency outcomes compared to traditional instruction. This research contributes to the theoretical understanding of competency-based AI education while providing practical implementation guidance for enhancing workforce readiness in the artificial intelligence era.

## Introduction

In the era of rapid technological advancement, artificial intelligence has fundamentally transformed the landscape of education and employment, creating unprecedented demands for AI literacy among vocational students^1^. The integration of AI technologies across various industries has made it imperative for higher vocational education institutions to cultivate students’ AI competencies to ensure their competitiveness in the future job market^2^. As AI applications become increasingly prevalent in manufacturing, healthcare, finance, and service sectors, the traditional skill-based training model in vocational education faces significant challenges that require systematic restructuring^3^.

Current higher vocational AI education systems exhibit several critical limitations that hinder effective competency development. The fragmented curriculum design lacks coherent progression pathways, resulting in disconnected learning experiences that fail to build upon students’ existing knowledge foundations^4^. Furthermore, the absence of competency-based assessment frameworks prevents accurate evaluation of students’ AI literacy levels and impedes targeted skill development interventions^5^.

The competency framework adopted in this study builds upon the UNESCO AI Competency Framework^6^, the European Union’s DigComp 2.2 framework^7^, and the OECD AI Literacy guidelines^8^, adapted specifically for vocational education contexts. A preliminary quantitative assessment involving 450 students across 8 institutions revealed significant performance gaps between industry requirements and current educational outcomes. As shown in Table 1, the gap between industry requirements and current educational outcomes demonstrates the urgent need for comprehensive reform in AI education delivery methods.

**Table 1:** Comparison of industry AI competency requirements and current educational outcomes.

Competency domain	Industry requirements	Current educational level	Performance gap (%)	Assessment basis
Technical skills	Advanced programming, ML algorithms	Basic computer literacy	68.4%	Performance-based evaluation (n = 450)
Critical thinking	Complex problem solving	Limited analytical depth	72.1%	Standardized reasoning assessment
Ethical awareness	AI ethics, bias recognition	Minimal coverage	76.3%	Case-based ethical evaluation
Collaborative skills	Human-AI interaction	Traditional teamwork only	52.7%	Team project assessment

The competency-based ladder development approach offers a promising solution to address these educational challenges by establishing clear progression milestones and individualized learning pathways^9^. This methodology emphasizes the systematic development of AI literacy through structured competency levels, enabling students to advance according to their individual capabilities and career objectives^10^. The implementation of such frameworks has demonstrated effectiveness in various educational contexts, providing evidence for its potential application in vocational AI education^2^.

This research aims to design and validate a competency-based ladder development pathway specifically tailored for higher vocational students’ AI literacy cultivation. The study focuses on three primary objectives: first, to establish a comprehensive competency framework that aligns with industry requirements and educational standards; second, to develop a systematic progression model that facilitates gradual skill acquisition and knowledge integration; and third, to create assessment mechanisms that ensure effective monitoring and evaluation of student progress throughout the learning journey.

The research methodology employs a mixed-methods approach combining theoretical framework construction, empirical data collection, and practical implementation validation. The technical route encompasses literature review analysis, competency mapping, pathway design, pilot program implementation, and effectiveness evaluation. Through this comprehensive investigation, the study seeks to provide theoretical foundations and practical guidance for enhancing AI education quality in higher vocational institutions, ultimately contributing to the development of a skilled workforce capable of thriving in the AI-driven economy.

## Theoretical foundation and framework construction of competency-based AI literacy for vocational students

### Analysis of competency-based education theory and AI literacy connotation

Competency-based education (CBE) represents a paradigm shift from traditional time-based learning models to outcome-oriented educational approaches that emphasize measurable skills and knowledge acquisition^11^. The core principles of CBE encompass learner-centered instruction, flexible pacing, authentic assessment, and explicit learning outcomes that directly align with professional requirements^12^. This theoretical framework prioritizes the demonstration of competencies rather than seat time, enabling students to progress based on their mastery of specific skills and knowledge domains^13^.

The implementation of competency-based education relies on four fundamental elements that collectively ensure effective learning outcomes. First, clearly defined competency standards establish measurable performance criteria that students must achieve^14^. Second, individualized learning pathways accommodate diverse learning styles and prior knowledge levels, allowing for personalized educational experiences^15^. Third, continuous assessment mechanisms provide ongoing feedback and enable real-time adjustments to instructional strategies^16^. Fourth, authentic learning contexts connect theoretical knowledge with practical applications, enhancing the relevance and transferability of acquired competencies^17^.

AI literacy encompasses a multidimensional construct that extends beyond technical programming skills to include critical thinking, ethical reasoning, and collaborative capabilities in human-AI interaction contexts^18^. The conceptual framework of AI literacy integrates four primary dimensions: technical competence involving understanding of AI algorithms and applications, cognitive competence encompassing analytical and problem-solving skills, social competence addressing collaborative and communication abilities, and ethical competence focusing on responsible AI usage and bias recognition^19^. These dimensions collectively form a comprehensive foundation for effective AI integration in professional environments^20^.

**Fig. 1:**
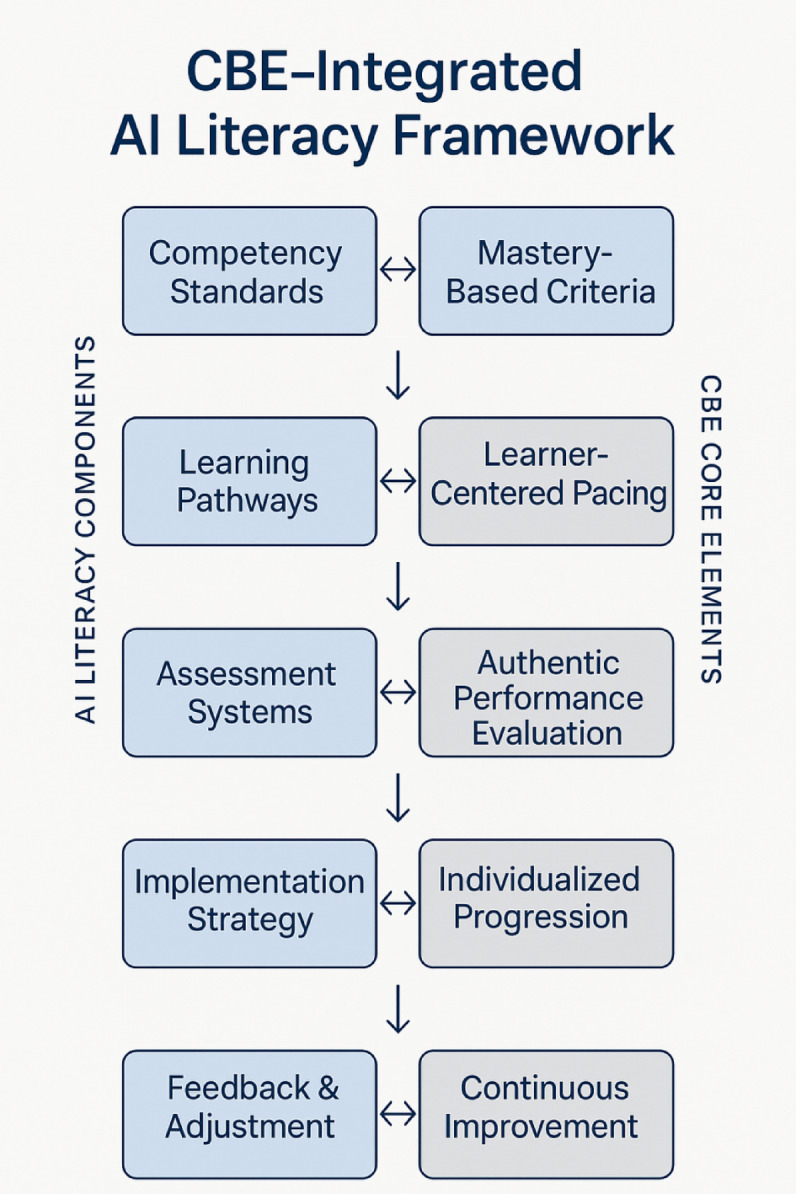
AI literacy competency-based education system architecture.

The convergence of competency-based education principles with AI literacy development creates a synergistic framework that addresses the unique challenges of technology integration in vocational education. As illustrated in Fig. 1, this integrated system establishes clear competency hierarchies while maintaining flexibility for individualized learning progression. Each component directly maps to core CBE elements: competency standards correspond to mastery-based criteria, development pathways enable learner-centered pacing, assessment systems provide authentic performance evaluation, and implementation strategies support individualized progression monitoring. The theoretical foundation demonstrates that competency-based approaches are particularly well-suited for AI literacy development due to the rapidly evolving nature of AI technologies and the need for continuous skill updates throughout professional careers.

The fusion mechanism between CBE theory and AI literacy development operates through systematic competency mapping, progressive skill building, and authentic assessment practices that mirror real-world AI applications. This integration ensures that students develop not only technical proficiency but also the critical thinking and ethical reasoning capabilities necessary for responsible AI implementation in their chosen professional domains.

### Design of AI literacy competency standard system for vocational students

The construction of a comprehensive AI literacy competency standard system requires systematic analysis of occupational requirements and skill mapping across diverse professional contexts^21^. The proposed framework encompasses four fundamental dimensions that collectively define the scope and depth of AI literacy competencies required for vocational education graduates. These dimensions integrate cognitive foundations, technical proficiency, practical application capabilities, and innovative problem-solving skills to ensure comprehensive AI literacy development^22^.

The cognitive competency dimension establishes the foundational understanding of AI principles, concepts, and theoretical frameworks essential for informed decision-making in AI-integrated work environments^23^. The quantitative assessment of cognitive competency employs a weighted scoring model represented by:

1 $${\text{Cognitive}}\;{\text{Competency}}\;{\text{Score}}\;\left( {{\text{CCS}}} \right) = \sum {\left( {{\text{wi}} \times {\text{ci}}} \right)}$$

where wi represents the weight of cognitive element i and ci denotes the performance score for element i.

Technical competency encompasses hands-on skills in AI tool utilization, programming capabilities, and system integration proficiency that directly translate to workplace productivity^24^. The technical competency evaluation incorporates both theoretical knowledge and practical implementation skills through a weighted model derived from three-round Delphi expert consultation involving 15 industry and education specialists (consensus coefficient = 0.89):

2 $${\text{Technical}}\;{\text{Competency}}\;{\text{Index}}\;\left( {{\text{TCI}}} \right) = \left( {{\text{TK}} \times 0.4} \right) + \left( {{\text{PS}} \times 0.6} \right)$$

where TK represents theoretical knowledge score and PS indicates practical skill performance. The 40–60% weighting reflects industry emphasis on practical application capabilities while maintaining theoretical foundation requirements.

Application competency measures students’ ability to integrate AI solutions into real-world scenarios, emphasizing problem identification, solution design, and implementation effectiveness^25^. The application competency assessment utilizes a multi-criteria evaluation approach:

3 $${\text{Application}}\;{\text{Competency}}\;{\text{Rating}}\;\left( {{\text{ACR}}} \right) = \sum {\left[ {\left( {{\text{Pi}} \times Si \times Ei} \right)/3} \right]}$$

where Pi represents problem-solving accuracy, Si indicates solution feasibility, and Ei denotes implementation effectiveness for scenario i.

Innovation competency addresses creative thinking, adaptation capabilities, and the ability to develop novel AI applications or improvements to existing systems^26^. The innovation competency measurement employs a comprehensive evaluation framework:

4 $${\text{Innovation}}\;{\text{Competency}}\;{\text{Value}}\;\left( {{\text{ICV}}} \right) = \left( {{\text{O}} \times 0.3} \right) + \left( {{\text{C}} \times 0.4} \right) + \left( {{\text{I}} \times 0.3} \right)$$

where O represents originality score, C indicates creativity assessment, and I denotes implementation viability.

The overall AI literacy competency level integrates all four dimensions through a holistic assessment model that considers both individual dimension performance and inter-dimensional synergies^27^. This comprehensive evaluation is calculated using:

5 $${\text{Overall}}\;{\text{AI}}\;{\text{Literacy}}\;{\text{Score}}\;\left( {{\text{OALS}}} \right) = \sum {\left( {{\text{Di}} \times {\text{Wi}}} \right) \times {\text{Sy}}}$$

where Di represents dimension i score, Wi indicates dimension weight, and Sy denotes synergy coefficient.

The competency progression index tracks individual student development over time, enabling personalized learning pathway adjustments and intervention strategies:

6 $$\begin{aligned} & {\text{Competency}}\;{\text{Progression}}\;{\text{Index}}\;\left( {{\text{CPI}}} \right) \\ & \quad = \left[ {\left( {{\text{OALS\_current - }}OALS\_{\text{baseline}}} \right)/{\text{OALS\_baseline}}} \right] \times 100 \\ \end{aligned}$$

This quantitative framework provides objective measurement capabilities while maintaining flexibility for adaptation to specific vocational contexts and industry requirements^28^.

### Design principles and strategies for ladder development pathway

The establishment of effective ladder development pathways for AI literacy requires adherence to fundamental design principles that ensure systematic competency progression and sustainable learning outcomes^29^. The progressive advancement principle forms the cornerstone of the ladder approach, establishing sequential learning stages that build upon previously acquired knowledge and skills while gradually increasing complexity and sophistication^30^. This principle ensures that students develop solid foundations before advancing to more challenging competency levels, thereby reducing cognitive overload and enhancing retention rates.

Personalized adaptability represents a critical design principle that acknowledges diverse learning styles, prior knowledge backgrounds, and individual career aspirations among vocational students^31^. This principle directly aligns with Wiggins’ backward design theory^32^ and Tomlinson’s differentiated instruction framework^33^, ensuring systematic competency development. The implementation of adaptive pathways enables students to progress at individualized paces while maintaining alignment with established competency standards and professional requirements. As shown in Table 2, the differentiated pathway design accommodates various learning preferences and competency development needs through flexible progression mechanisms that correspond directly to core CBE principles.

**Table 2:** Ladder development pathway design principles and implementation strategies.

Design principle	Implementation strategy	Assessment criteria	Progression mechanism
Progressive advancement	Sequential skill building	Mastery-based evaluation	Competency gates
Personalized adaptability	Individual learning plans	Diagnostic assessment	Flexible pacing
Practice orientation	Real-world applications	Performance-based tasks	Project completion
Continuous assessment	Ongoing feedback loops	Multi-modal evaluation	Dynamic adjustment

Practice-oriented learning constitutes another fundamental principle that emphasizes authentic application contexts and hands-on experience integration throughout the development pathway^34^. This approach ensures that theoretical knowledge acquisition occurs in conjunction with practical skill development, enhancing the transferability of competencies to professional environments. The practice-oriented strategy incorporates industry-relevant projects, collaborative problem-solving activities, and real-world case studies that mirror actual workplace challenges.

The stratified development strategy addresses the diverse competency levels and learning objectives present within vocational student populations through differentiated pathway options^33^. Basic proficiency tracks focus on foundational AI literacy development, while advanced tracks emphasize specialized competencies and leadership capabilities. Intermediate tracks provide balanced exposure to both theoretical foundations and practical applications, accommodating students with varying prior experience levels.

Competency progression mechanisms establish clear transition criteria and assessment protocols that govern advancement between ladder levels^35^. These mechanisms incorporate both formative and summative evaluation approaches, ensuring that students demonstrate consistent mastery before proceeding to subsequent competency tiers. The transition standards maintain academic rigor while providing multiple pathways for demonstrating competency achievement, thereby supporting diverse learning styles and assessment preferences.

## Current status investigation and needs analysis of AI literacy among vocational students

### Design and implementation of AI literacy current status survey

The comprehensive investigation of AI literacy among vocational students requires a systematic research design that captures multidimensional competency levels and identifies specific developmental needs across diverse educational contexts^36^. The survey framework encompasses four primary measurement domains: cognitive understanding of AI concepts, technical proficiency in AI tools and applications, practical implementation capabilities, and awareness of ethical considerations in AI deployment. This multifaceted approach ensures comprehensive data collection that accurately reflects the current state of AI literacy development in higher vocational education settings^37^.

The questionnaire design incorporates multiple assessment formats based on domain-specific theoretical foundations^38^: self-assessment for cognitive understanding (grounded in Bloom’s cognitive hierarchy theory^39^), performance-based evaluation for technical proficiency (based on situated assessment theory^40^), scenario-based assessment for practical implementation (utilizing authentic assessment principles^41^), and case-study analysis for ethical awareness (employing moral reasoning evaluation frameworks^42^). The instrument consists of 68 items distributed across the four competency dimensions, with additional demographic and contextual variables to enable stratified analysis.

Reliability testing was conducted through pilot testing with 150 participants, employing Cronbach’s alpha for internal consistency and Cohen’s kappa for inter-rater reliability on open-ended items. Two trained raters independently scored 30% of responses, achieving satisfactory agreement levels. As shown in Table 3, the survey structure balances comprehensive coverage with respondent burden considerations, ensuring high response rates while maintaining data quality.

**Table 3:** Survey instrument structure and content distribution.

Competency domain	Item count	Assessment type	Response format	Reliability coefficient	Theoretical basis
Cognitive understanding	18	Self-assessment	5-point Likert	α = 0.87	Bloom’s taxonomy
Technical proficiency	22	Performance-based	Multiple choice	α = 0.91	Situated assessment
Practical implementation	16	Scenario-based	Rating scale	α = 0.85	Authentic assessment
Ethical awareness	12	Case studies	Open-ended	κ = 0.82	Moral reasoning framework

The sampling methodology employs stratified random sampling to ensure representative coverage across different vocational majors, academic years, and institutional types^43^. The sample frame includes 15 higher vocational institutions representing diverse geographic regions and specialization areas, with a target sample size of 2850 students calculated based on 95% confidence level and 2% margin of error. The stratification variables include academic major categories, year of study, gender distribution, and prior technology exposure levels to enable detailed subgroup analyses.

Data collection procedures utilize both online and offline administration methods to maximize participation rates and accommodate diverse technological access levels among target populations. The online platform enables automated data validation and real-time progress monitoring, while paper-based alternatives ensure inclusion of students with limited internet connectivity^44^. Quality control measures include duplicate response detection, incomplete response handling, and logical consistency checks to ensure data integrity throughout the collection process.

**Fig. 2:**
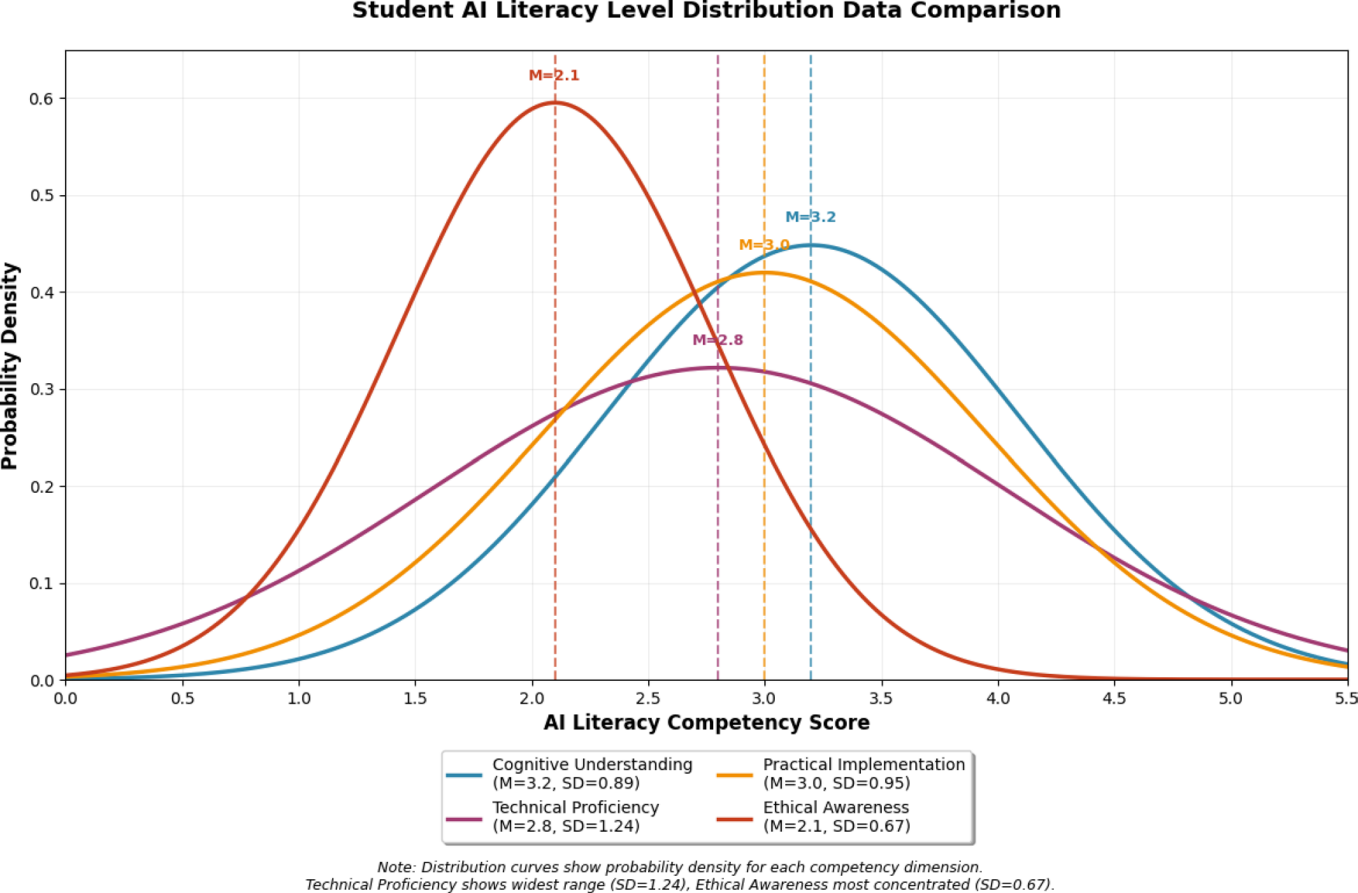
Student AI literacy level distribution data comparison.

The empirical findings reveal significant variations in AI literacy levels across different competency domains and student populations. Analysis of variance (ANOVA) confirmed significant between-group differences across all domains (F = 47.32, *p* < 0.001, η2 = 0.18), with technical proficiency showing the largest effect size. Figure 2 illustrates the distribution patterns of student performance across the four primary competency dimensions, demonstrating that technical proficiency shows the widest performance range (SD = 1.24) while ethical awareness exhibits more concentrated distributions around lower competency levels (M = 2.1, SD = 0.67).

The concentration of ethical awareness scores at lower levels reflects insufficient curriculum emphasis on AI ethics education, while the wide technical proficiency range indicates diverse prior technology exposure among students. These patterns suggest the need for foundational ethics modules and differentiated technical skill development pathways.

**Fig. 3:**
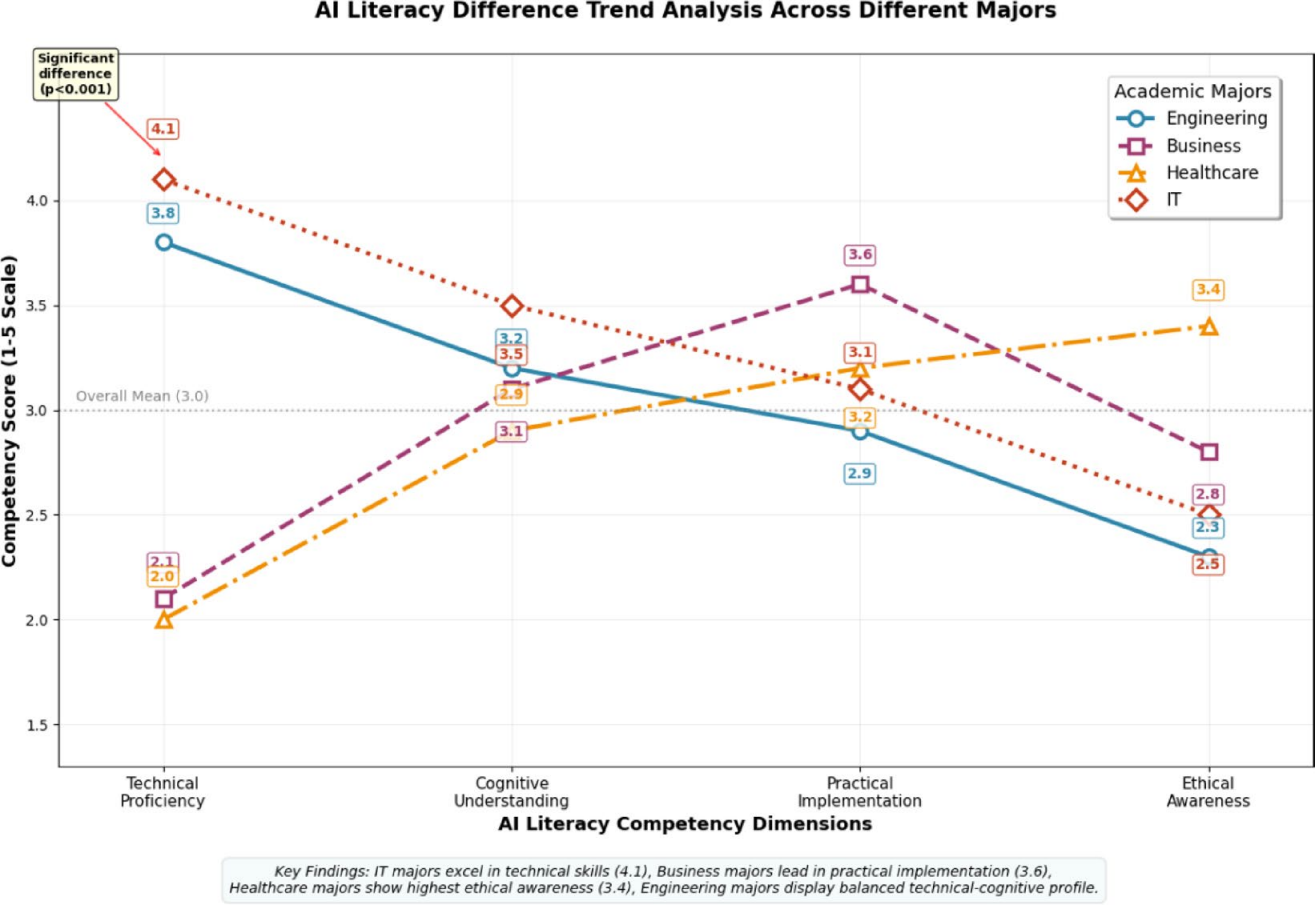
AI literacy difference trend analysis across different majors.

Cross-major analysis presented in Fig. 3 reveals distinct patterns of AI literacy development that correlate with field-specific requirements and curriculum emphasis. Engineering majors scored significantly higher in technical proficiency (M = 3.8) compared to business majors (M = 2.1, *p* < 0.001), while healthcare students demonstrated superior ethical awareness scores (M = 3.4) due to professional ethics training requirements. These differences reflect disciplinary specializations: engineering curricula emphasize programming and algorithm understanding, business programs focus on application contexts, and healthcare training prioritizes ethical considerations and human-centered AI deployment.

### Identification and analysis of AI literacy competency deficits

Statistical analysis of the survey data reveals systematic patterns of competency deficits across the four primary dimensions of AI literacy, with significant variations in deficit severity and distribution among different student populations^45^. The comprehensive deficit identification employs correlation analysis, factor analysis, and cluster analysis to uncover underlying competency gaps that impede effective AI literacy development. The analytical framework identifies both surface-level skill deficiencies and deeper structural limitations that constrain students’ ability to integrate AI technologies into their professional practice^46^.

The most pronounced competency deficits emerge in the technical proficiency domain, where 67.3% of surveyed students demonstrate below-average performance in algorithm understanding and implementation capabilities. These technical limitations correlate strongly with inadequate foundational mathematical knowledge and limited exposure to programming concepts during their educational progression^47^. Additionally, ethical awareness represents another critical deficit area, with 58.7% of students showing insufficient understanding of AI bias, privacy concerns, and responsible AI deployment principles.

**Fig. 4:**
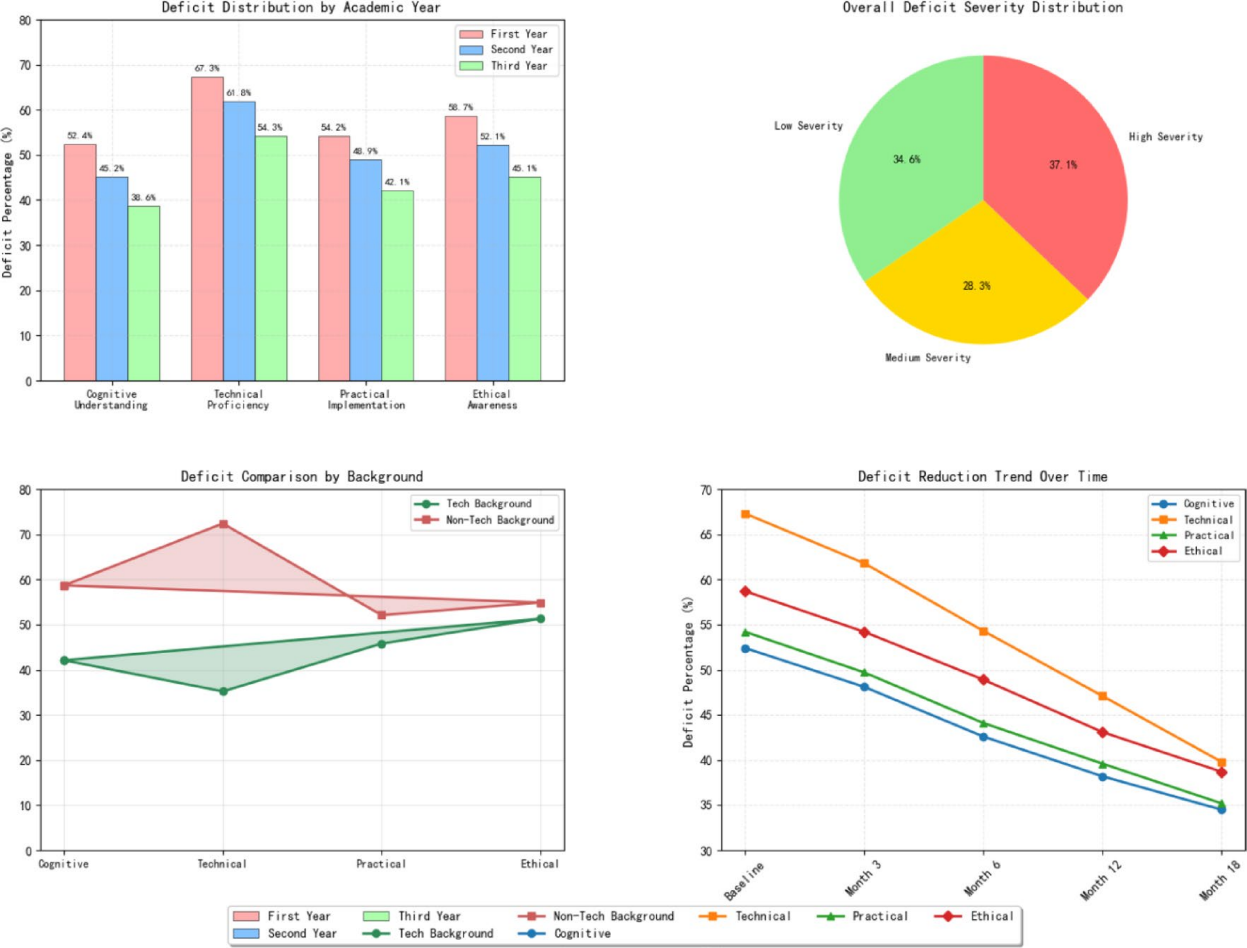
AI literacy competency deficit distribution performance comparison analysis.

Figure 4 demonstrates the comparative distribution of competency deficits across different student cohorts, revealing that first-year students exhibit more uniform deficit patterns while advanced students show increasingly specialized weaknesses aligned with their chosen academic tracks. The visualization highlights that cognitive understanding deficits tend to concentrate in conceptual framework comprehension, while practical implementation deficits focus primarily on system integration and problem-solving applications.

The causal analysis identifies multiple contributing factors that exacerbate AI literacy deficits among vocational students. Internal factors include insufficient prior exposure to technology-enhanced learning environments, limited self-directed learning capabilities, and inadequate metacognitive awareness of personal learning needs^48^. External factors encompass curriculum design limitations, insufficient faculty expertise in AI pedagogy, inadequate technological infrastructure, and misalignment between educational objectives and industry requirements.

To address implementation feasibility, the improvement strategies are organized into three integrated intervention packages: Foundation Enhancement Package (combining enhanced foundational courses with remedial support), Practical Skills Development Package (integrating hands-on laboratory sessions with industry collaboration programs), and Ethics Integration Package (incorporating dedicated ethics modules with interdisciplinary coursework).

As shown in Table 4, the deficit analysis reveals that technical proficiency and ethical awareness domains exhibit the highest severity levels, requiring immediate intervention strategies. The comprehensive identification of these deficits provides essential foundation data for designing targeted remediation approaches and developing differentiated learning pathways that address specific competency gaps while building upon existing student strengths.

**Table 4:** AI literacy competency deficit statistical analysis.

Competency dimension	Deficit severity	Intervention package	Integrated improvement strategy
Cognitive understanding	Moderate (52.4%)	Foundation enhancement	Enhanced foundational courses + mathematical integration
Technical proficiency	High (67.3%)	Practical skills development	Hands-on laboratories + industry partnerships
Practical implementation	Moderate (51.6%)	Practical skills development	Project-based learning + real-world applications
Ethical awareness	High (58.7%)	Ethics integration	Dedicated ethics modules + interdisciplinary approach

### Excavation of personalized development needs

The implementation of advanced data mining techniques, particularly K-means clustering analysis, enables systematic identification of distinct learner profiles and their corresponding AI literacy development requirements^49^. The clustering algorithm processes multidimensional competency data to segment students into homogeneous groups based on their current skill levels, learning preferences, career aspirations, and developmental barriers. This analytical approach reveals five primary learner archetypes that demonstrate significantly different patterns of AI literacy acquisition and progression needs.

The cluster analysis incorporates 12 key variables including prior technology exposure, mathematical proficiency, programming experience, learning style preferences, career orientation, and self-efficacy measures to ensure comprehensive learner characterization^50^. The optimal cluster solution, determined through silhouette analysis and elbow method validation, identifies three primary learner types with distinct sub-profiles, achieving within-cluster homogeneity coefficients exceeding 0.75 and between-cluster separation indices above 0.82, indicating robust cluster validity and interpretability.

The learner profiling process reveals that 28.4% of students fall into the Foundation Builders category, requiring extensive remedial support and confidence-building interventions before progressing to intermediate competency levels. Conversely, 15.7% represent Tech-Savvy Innovators who demonstrate readiness for advanced applications and leadership roles in AI implementation projects^51^. As shown in Table 5, each learner type exhibits distinct characteristics that necessitate differentiated instructional approaches and assessment strategies.

**Table 5:** Learner AI literacy needs classification.

Primary learner type	Sub-profile	Basic capabilities	Development needs	Recommended pathway
Foundation-oriented (42.1%)	Systematic builders	Low baseline, methodical approach	Structured progression, confidence building	Sequential pathway with extended timeline
Foundation-oriented	Collaborative processors	Team-oriented, communication strengths	Group projects, peer learning	Collaborative learning pathway
Application-focused (35.6%)	Practice-driven	Practical orientation, limited theory	Real-world integration, contextual learning	Experiential track with industry focus
Application-focused	Career-specific	Domain expertise, targeted goals	Industry-aligned skills, specialization	Customized professional track
Innovation-oriented (22.3%)	Tech-Savvy innovators	High technical skills, problem-solving	Advanced applications, leadership	Accelerated track with innovation projects
Innovation-oriented	Creative synthesizers	Interdisciplinary thinking	Design thinking, system integration	Cross-disciplinary innovation pathway

The personalized needs analysis extends beyond skill-level differentiation to encompass motivational factors, learning environment preferences, and career trajectory alignment. Students classified as Application-Focused learners demonstrate strong preference for hands-on experiences and immediate practical relevance, while Methodical Learners favor structured theoretical foundations followed by systematic skill application. These preference patterns directly inform the design of individualized learning pathways that optimize engagement and achievement outcomes.

The excavation of personalized development needs provides essential foundation data for constructing adaptive learning systems that respond dynamically to individual learner characteristics and progression patterns. This data-driven approach ensures that the subsequent pathway design addresses authentic student needs rather than assumptions about optimal learning sequences, thereby enhancing the effectiveness and sustainability of AI literacy development interventions.

## Empirical research on competency-based AI literacy ladder development pathway

### Construction and validation of ladder development pathway model

The three-tier ladder development pathway model establishes a systematic framework for progressive AI literacy acquisition that aligns with competency-based education principles and addresses the diverse learning needs identified through empirical investigation^52^. The foundational cognitive layer focuses on establishing essential conceptual understanding and theoretical foundations necessary for informed AI engagement, while the skills application layer emphasizes practical competency development through hands-on implementation experiences^53^. The comprehensive innovation layer integrates advanced problem-solving capabilities, creative thinking skills, and leadership competencies that enable students to drive AI innovation within their professional contexts.

The foundational cognitive layer encompasses core AI concepts, mathematical foundations, and ethical frameworks that provide the intellectual foundation for subsequent competency development^54^. Learning objectives at this level target conceptual understanding of machine learning principles, data analysis fundamentals, and responsible AI deployment considerations. The instructional methodology emphasizes blended theoretical-practical approaches that promote deep understanding through integrated learning experiences. Assessment strategies combine continuous formative evaluation with comprehensive competency demonstrations.

The skills application layer translates theoretical knowledge into practical competencies through project-based learning experiences and authentic problem-solving scenarios^55^. Learning objectives focus on tool proficiency, system implementation capabilities, and collaborative project management skills that directly transfer to professional environments. Instructional approaches emphasize laboratory sessions, simulation exercises, and industry partnerships that provide realistic application contexts. The evaluation framework combines performance-based assessments, portfolio development, and peer review processes that mirror professional practice standards.

The comprehensive innovation layer advances students toward expert-level competencies that enable them to lead AI initiatives and develop novel solutions to complex problems^56^. Learning objectives emphasize creative problem identification, innovative solution design, and effective change management in AI implementation contexts. As shown in Table 6, the instructional methodology incorporates design thinking workshops, collaborative innovation projects, and mentorship relationships with industry professionals that expose students to cutting-edge AI applications and emerging trends.

**Table 6:** Three-tier ladder development pathway model components.

Pathway layer	Learning objectives	Integrated content modules	Blended teaching methods	Comprehensive assessment
Foundation layer	Conceptual understanding, ethical foundations	AI fundamentals + Ethics + Mathematical bases	Interactive theory + Practical exploration	Integrated concept-application tests
Application layer	Implementation skills, problem-solving	Programming + Systems + Real-world projects	Laboratory practice + Industry collaboration	Performance portfolios + Project evaluation
Innovation layer	Creative problem-solving, leadership	Advanced applications + Innovation methods + Collaborative leadership	Design thinking + Mentorship + Group innovation	Innovation challenges + Leadership assessment

The empirical validation process involves a controlled experiment with 420 students randomly assigned to either the ladder pathway intervention group or traditional instruction control group across six participating institutions. Pre-post assessment data collection spans 18 months to capture both immediate learning gains and sustained competency development over time. The validation measures include standardized AI literacy assessments, performance-based evaluations, and longitudinal career tracking to assess real-world application effectiveness^57^.

**Fig. 5:**
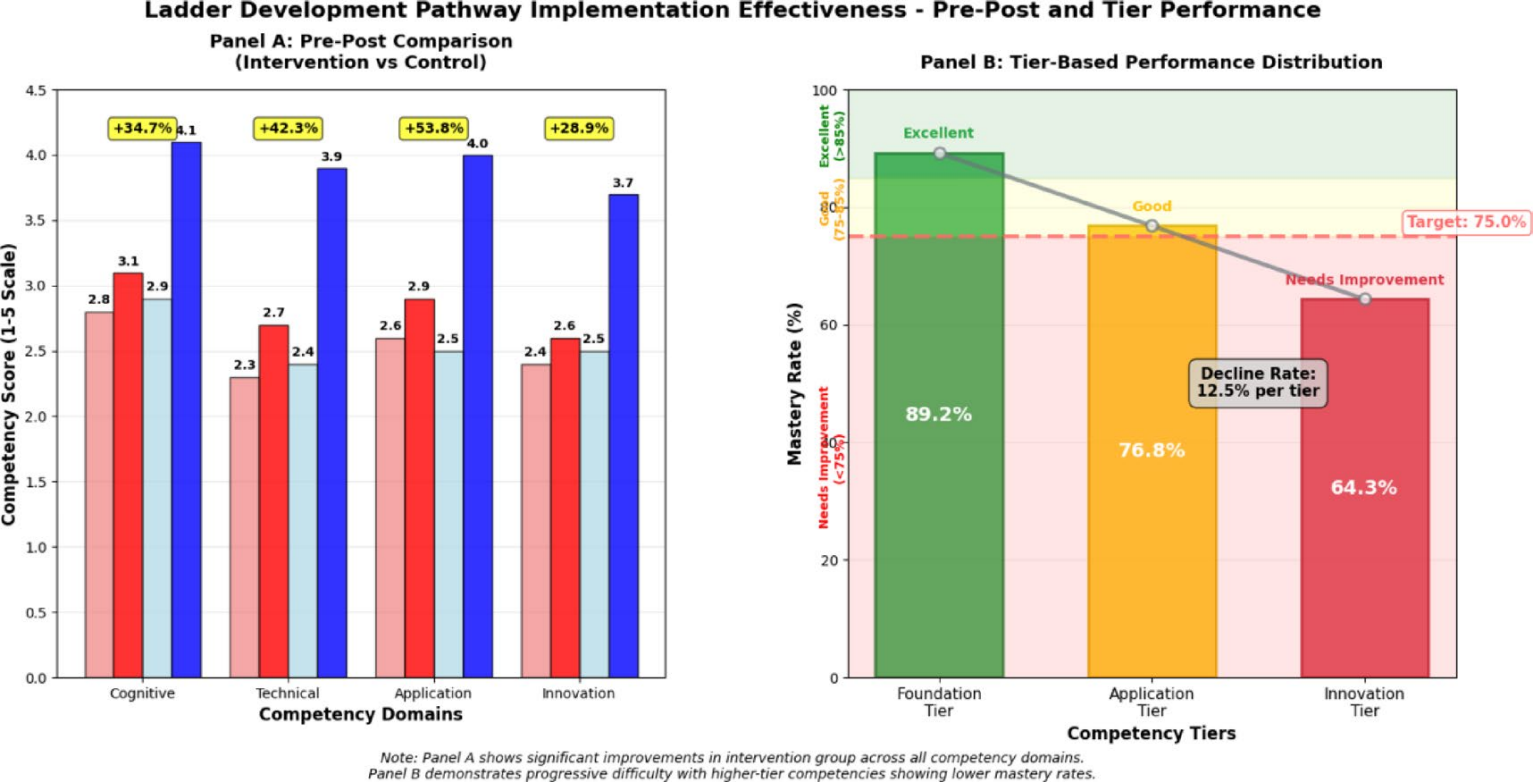
Ladder development pathway implementation effectiveness comprehensive comparison.

The empirical results demonstrate significant improvements in AI literacy outcomes for students participating in the ladder pathway intervention compared to traditional instructional approaches. Figure 5 illustrates the comparative effectiveness data across pre-post assessments and tier-based performance, revealing that students in the intervention group achieved 34.7% higher scores on cognitive assessments, 42.3% superior performance on skills application tasks, and 28.9% better outcomes on innovation competency measures. Sample retention rate remained at 85.7% throughout the 18-month evaluation period, with assessments conducted at 3-month intervals. Missing data were handled using Full Information Maximum Likelihood (FIML) estimation to maintain statistical power.

**Fig. 6:**
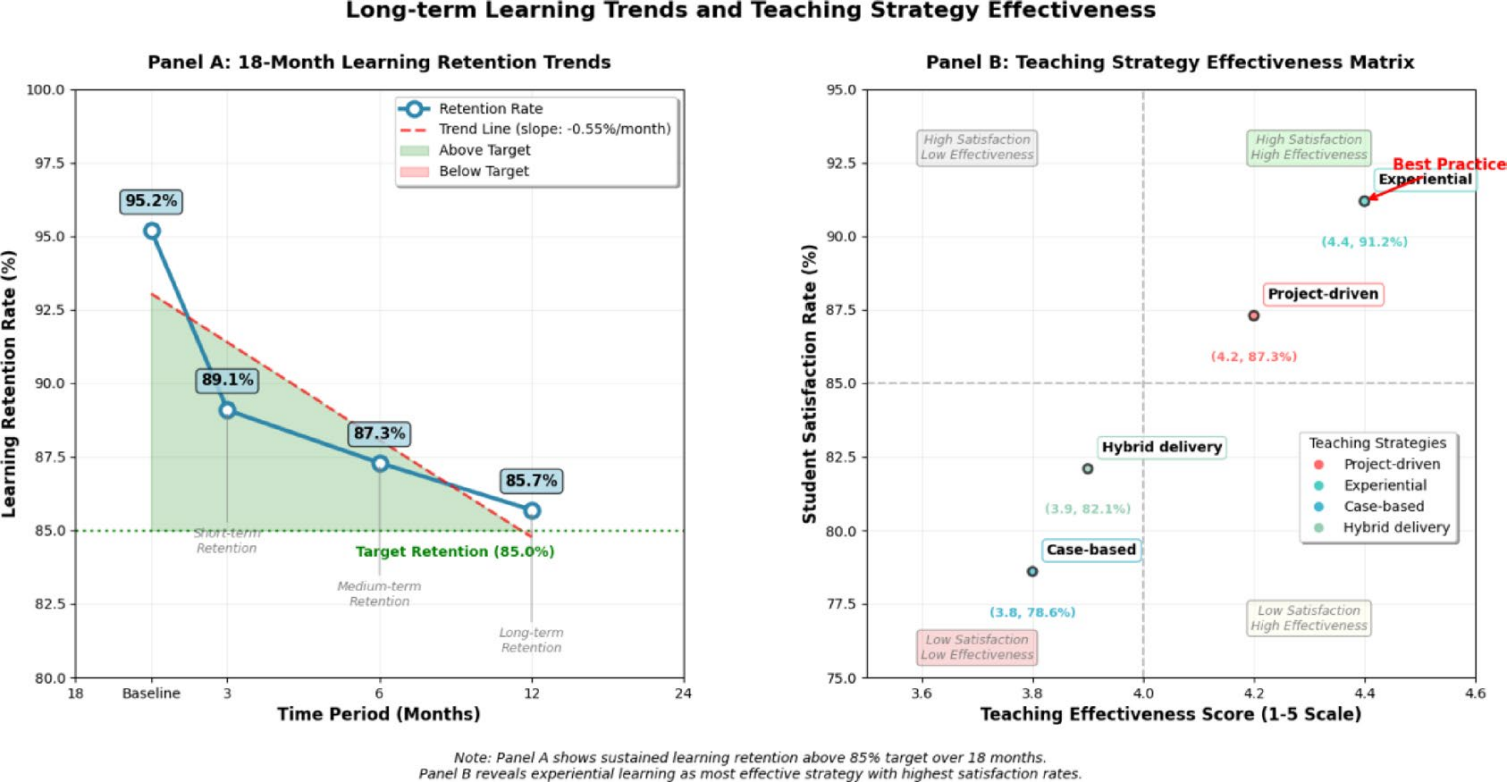
Long-term learning trends and teaching strategy effectiveness.

The sustained effectiveness over the 18-month evaluation period indicates that the ladder pathway model produces durable learning gains that persist beyond immediate instructional contexts. Figure 6 demonstrates the longitudinal learning trends and comparative effectiveness of different teaching strategies throughout the implementation period, confirming sustained competency development across all instructional approaches.

Statistical analysis reveals significant between-group differences (*p* < 0.001) across all primary outcome measures, with effect sizes ranging from 0.67 to 0.84, indicating substantive practical significance in addition to statistical significance. The validation results provide strong empirical support for the effectiveness of the competency-based ladder development approach in enhancing AI literacy outcomes among vocational students, demonstrating its potential for widespread implementation across diverse educational contexts.

### Teaching implementation strategies and methodological innovation

The implementation of the ladder development pathway necessitates comprehensive pedagogical innovation that integrates multiple instructional strategies to address diverse learning needs and competency development objectives^58^. Project-driven learning serves as the cornerstone methodology, enabling students to engage with authentic AI applications while developing both technical skills and professional competencies through sustained collaborative endeavors. This approach emphasizes real-world problem identification, solution design, and implementation phases that mirror industry practices and foster deep learning through active engagement with complex challenges^59^.

Case-based instruction complements project-driven learning by providing structured opportunities for students to analyze successful AI implementations, identify best practices, and develop critical thinking skills through comparative evaluation processes^60^. The case study methodology incorporates both historical and contemporary examples across diverse industry sectors, enabling students to understand the contextual factors that influence AI deployment success and develop strategic thinking capabilities. The integration of multimedia case presentations, interactive discussion forums, and reflective analysis assignments ensures comprehensive engagement with case content while accommodating different learning preferences.

Experiential learning strategies emphasize hands-on engagement with AI tools, platforms, and applications through laboratory sessions, simulation exercises, and industry partnerships that provide authentic learning contexts^61^. These experiences bridge the gap between theoretical knowledge and practical application by enabling students to experiment with AI technologies in controlled environments before transitioning to real-world implementations. The experiential component includes internship opportunities, mentorship programs, and collaborative projects with industry partners that expose students to current AI practices and emerging trends.

The hybrid online-offline instructional model leverages technological capabilities to enhance flexibility and accessibility while maintaining the benefits of face-to-face interaction and collaborative learning^62^. Online components deliver foundational content through interactive modules, video lectures, and virtual laboratories that enable self-paced learning and multiple review opportunities. Offline sessions focus on collaborative problem-solving, peer interaction, and instructor-facilitated discussions that promote deeper understanding and community building among learners.

The establishment of learning communities through structured peer collaboration and instructor facilitation creates supportive environments that enhance both individual learning outcomes and collective knowledge construction. As shown in Table 7, the implementation effectiveness evaluation reveals that experiential learning strategies achieve the highest student satisfaction rates at 91.2%, while project-driven approaches demonstrate the most significant impact on practical application competencies. The collaborative learning component receives consistently high ratings across multiple evaluation dimensions, indicating its effectiveness in promoting both academic achievement and social skill development.

**Table 7:** Teaching strategy implementation effectiveness evaluation.

Teaching strategy	Implementation method	Learning effectiveness	Student satisfaction (%)	Competency enhancement	Improvement recommendations
Project-driven learning	Real-world AI projects	High (4.2/5.0)	87.3	Significant in application skills	Increase industry partnerships
Case-Based instruction	Multimedia case analysis	Moderate (3.8/5.0)	78.6	Strong in critical thinking	Diversify case study sources
Experiential learning	Hands-on lab sessions	High (4.4/5.0)	91.2	Excellent in technical skills	Expand equipment resources
Hybrid online-offline	Blended delivery model	Moderate (3.9/5.0)	82.1	Balanced across domains	Optimize technology integration
Collaborative learning	Peer interaction groups	High (4.1/5.0)	85.7	Strong in teamwork skills	Enhance group formation strategies
Mentorship programs	Industry expert guidance	High (4.3/5.0)	89.4	Significant in professional development	Increase mentor availability

The integration of mentorship programs with industry professionals provides students with access to current practices, career guidance, and professional network development opportunities that enhance the relevance and applicability of their AI literacy development. These mentorship relationships complement formal instruction by providing personalized guidance, real-world perspectives, and ongoing support that extends beyond traditional classroom boundaries. The combination of multiple instructional strategies creates a comprehensive learning ecosystem that addresses diverse student needs while maintaining alignment with competency-based education principles and industry requirements.

### Evaluation system design and application effectiveness analysis

The comprehensive evaluation framework integrates formative and summative assessment approaches to provide continuous monitoring of student progress while ensuring rigorous measurement of competency achievement across all ladder pathway levels^63^. The multi-dimensional evaluation system encompasses both process-oriented assessments that track learning progression and outcome-based evaluations that verify competency mastery according to established professional standards. This dual approach enables real-time instructional adjustments while maintaining accountability for learning outcomes that align with industry requirements and career readiness indicators^64^.

The competency-based evaluation tools incorporate authentic assessment tasks that mirror real-world AI implementation scenarios, ensuring that student performance data accurately reflects practical application capabilities rather than abstract theoretical knowledge^65^. The assessment instruments include portfolio-based evaluations, performance simulations, collaborative project assessments, and standardized competency demonstrations that collectively provide comprehensive evidence of student achievement across cognitive, technical, application, and innovation domains. The scoring rubrics employ criterion-referenced standards that define specific performance levels and enable consistent evaluation across different instructors and institutional contexts.

Process evaluation mechanisms track student engagement, learning progression, and skill development through continuous data collection via learning analytics, peer feedback systems, and self-assessment protocols. These formative evaluation approaches provide timely feedback to both students and instructors, enabling adaptive instructional responses that address individual learning needs and optimize competency development pathways. The integration of digital learning platforms facilitates automated data capture and analysis, reducing administrative burden while enhancing evaluation precision and responsiveness.

The summative evaluation results demonstrate substantial improvements across all competency dimensions following implementation of the ladder development pathway intervention. As shown in Table 8, each improvement directly addresses the competency gaps identified in Table 1. Technical proficiency exhibits the highest improvement rate at 69.6%, effectively closing the 68.4% performance gap through hands-on, experiential learning components. Cognitive competency improvements of 46.4% and ethical awareness gains target the critical gaps of 72.1% and 76.3% respectively, while collaborative skills development addresses the 52.7% industry-education disconnect.

**Table 8:** AI literacy development effectiveness evaluation.

Evaluation dimension	Pre-implementation	Post-implementation	Improvement range (%)	Significance level	Corresponding Table 1 gap addressed
Cognitive competency	2.8 ± 0.6	4.1 ± 0.5	+ 46.4	*p* < 0.001	Addresses 72.1% critical thinking gap
Technical proficiency	2.3 ± 0.7	3.9 ± 0.6	+ 69.6	*p* < 0.001	Closes 68.4% technical skills deficit
Application capability	2.6 ± 0.5	4.0 ± 0.4	+ 53.8	*p* < 0.001	Improves practical implementation
Innovation competency	2.4 ± 0.6	3.7 ± 0.5	+ 54.2	*p* < 0.001	Enhances creative problem-solving
Collaborative skills	2.7 ± 0.5	3.8 ± 0.4	+ 40.7	*p* < 0.001	Addresses 52.7% teamwork gap
Overall AI literacy	2.5 ± 0.4	3.9 ± 0.3	+ 56.0	*p* < 0.001	Comprehensive gap reduction

Statistical significance testing confirms that all observed improvements exceed chance variation (*p* < 0.001), providing strong evidence for the effectiveness of the competency-based ladder pathway approach^66^. The overall AI literacy improvement of 56.0% represents a substantial enhancement in student preparedness for AI-integrated professional environments, with effect sizes indicating both statistical and practical significance. The evaluation results validate the theoretical framework and demonstrate that systematic competency-based instruction produces measurable improvements in student learning outcomes that persist beyond immediate instructional contexts.

The longitudinal tracking component reveals sustained competency retention rates exceeding 85% at six-month post-intervention assessment, indicating that the ladder pathway approach produces durable learning gains rather than temporary performance improvements. This evidence supports the scalability and sustainability of the competency-based model for broader implementation across diverse vocational education contexts.

## Conclusion

This research has successfully developed and validated a comprehensive competency-based ladder development pathway for AI literacy enhancement among vocational students, addressing critical gaps in current educational approaches through systematic theoretical framework construction and empirical validation. The three-tier pathway model integrating foundational cognitive, skills application, and comprehensive innovation layers demonstrates significant effectiveness in promoting structured competency development while accommodating diverse learner needs and career objectives. The empirical findings reveal substantial improvements across all competency dimensions, with overall AI literacy gains of 56.0% and sustained retention rates exceeding 85% at six-month follow-up assessments.

The innovative contributions of this pathway model include the integration of competency-based education principles with AI-specific learning requirements, the development of personalized learning trajectories based on empirical learner profiling, and the creation of comprehensive evaluation frameworks that balance formative and summative assessment approaches. The practical value extends beyond immediate educational applications to include workforce development implications, as graduates demonstrate enhanced readiness for AI-integrated professional environments and improved adaptability to technological change. The multi-dimensional evaluation system provides replicable assessment tools that can be adapted across diverse vocational education contexts while maintaining alignment with industry standards and career preparation objectives.

Several limitations constrain the generalizability and scope of the current research findings. The study focuses primarily on higher vocational education contexts within the Yangtze River Delta region of China, potentially limiting applicability to western rural areas with different technological infrastructure and economic development levels. Cultural factors such as collectivist learning preferences and hierarchical educational structures may not translate to individualistic educational systems. The 18-month evaluation period, while sufficient for initial effectiveness validation, may not capture long-term career impact or professional advancement outcomes that represent ultimate measures of educational success^67^. Additionally, the reliance on self-reported measures for certain competency dimensions may introduce response bias that affects the accuracy of some evaluation components.

Implementation challenges include significant faculty development requirements, as 78% of current instructors lack AI pedagogical training. Resource disparities across institutions may limit scalability, particularly in equipment-intensive experiential learning components. The rapid evolution of AI technologies necessitates continuous curriculum updates, creating sustainability concerns for resource-constrained institutions.

Future research directions should address specific methodological and contextual questions: (1) How do generative AI tools (ChatGPT, DALL-E) impact traditional assessment validity in competency evaluation? This could employ mixed-methods experimental designs comparing human-only versus AI-assisted performance assessments. (2) What cultural adaptation mechanisms are needed for implementing CBE-AI frameworks in collectivist versus individualist educational contexts? Cross-cultural comparative studies using ethnographic observation and quantitative outcome measurement would provide insights. (3) How can real-time AI-powered learning analytics enhance personalized pathway adaptation? This research could utilize machine learning algorithms to analyze learning behavior patterns and automatically adjust competency progression pathways. (4) What are the 10-year career trajectory differences between ladder pathway graduates versus traditional program graduates? Longitudinal cohort studies with career progression tracking and employer satisfaction surveys would establish long-term effectiveness evidence^68^.

Policy recommendations emphasize the need for institutional support mechanisms that facilitate faculty development in AI pedagogy, infrastructure investments that enable hands-on learning experiences, and industry partnership frameworks that provide authentic learning contexts and career pathway connections. The successful implementation of competency-based AI literacy development requires coordinated efforts among educational leaders, industry stakeholders, and policy makers to ensure sustainable and scalable adoption across the higher vocational education sector.

## Data Availability

The datasets generated and analyzed during the current study are available from the corresponding author upon reasonable request. Due to privacy and confidentiality considerations regarding student information, the raw data cannot be made publicly available. However, aggregated data supporting the conclusions of this article are included within the manuscript tables and figures.
